# Diagnostic Significance of Metagenomic Next-Generation Sequencing for Community-Acquired Pneumonia in Southern China

**DOI:** 10.3389/fmed.2022.807174

**Published:** 2022-02-15

**Authors:** Hanying Liu, Ying Zhang, Guiyang Chen, Shenghua Sun, Jiangang Wang, Fengyi Chen, Chun Liu, Quan Zhuang

**Affiliations:** ^1^Department of Respiratory Diseases, The 3rd Xiangya Hospital, Central South University, Changsha, China; ^2^Transplantation Center, The 3rd Xiangya Hospital, Central South University, Changsha, China; ^3^Department of Cardiology, Hunan Aerospace Hospital, Changsha, China; ^4^Department of Health Management, The 3rd Xiangya Hospital, Central South University, Changsha, China; ^5^Vision Medicals Co. Ltd, Guangzhou, China; ^6^Research Center of National Health Ministry on Transplantation Medicine, Changsha, China

**Keywords:** mNGS, pneumonia, diagnostic significance, pathogen, BALF

## Abstract

**Background:**

The morbidity and mortality of community-acquired pneumonia are relatively high, but many pneumonia pathogens cannot be identified accurately. As a new pathogen detection technology, metagenomic next-generation sequencing (mNGS) has been applied more and more clinically. We aimed to evaluate the diagnostic significance of mNGS for community-acquired pneumonia (CAP) in the south of China.

**Methods:**

Our study selected CAP patients who visited the 3rd Xiangya Hospital from May 2019 to April 2021. Pathogens in bronchoalveolar lavage fluid (BALF) specimens were detected using mNGS and traditional microbiological culture. mNGS group: detected by both mNGS and BALF culture; control group: detected only by BALF or sputum culture. The diagnostic performance of pathogens and the antibiotic adjustments were compared within mNGS group.

**Results:**

The incidence of acute respiratory distress syndrome (ARDS) was 28.3% in the mNGS group and 17.3% in the control group. Within the mNGS group, the positive rate of pathogens detected by mNGS was 64%, thus by BALF culture was only 28%. Pathogens detected by mNGS were consisted of bacteria (55%), fungi (18%), special pathogens (18%), and viruses (9%). The most detected pathogen by mNGS was *Chlamydia psittaci*. Among the pathogen-positive cases, 26% was not pathogen-covered by empirical antibiotics, so most of which were made an antibiotic adjustment.

**Conclusions:**

mNGS can detect pathogens in a more timely and accurate manner and assist clinicians to adjust antibiotics in time. Therefore, we recommend mNGS as the complementary diagnosis of severe pneumonia or complicated infections.

## Introduction

Community-acquired pneumonia (CAP) is a common disease with high mortality (1). According to the clinical phenotype, the pathogens for up to 60% of infectious diseases were still unknown (2, 3), and the mortality of CAP in need of emergency treatment exceeds 40% (4). Pathogens that cause pneumonia include common bacteria (such as *Streptococcus pneumoniae*), fungi, viruses, and some atypical pathogens such as *Mycoplasma pneumoniae, Chlamydia*, and *Legionella* (1). In a study of 329 clinical samples, it was found that the main pathogens of patients with different immune status were diverse. Among patients with normal immunity, the pathogens are mainly *S. pneumoniae, rhinovirus*, and *influenza*. The main pathogens in immunocompromised patients are *Pneumocystis, Klebsiella pneumonia, S. pneumoniae, Haemophilus influenza*, and *Pseudomonas aeruginosa* (5). In recent years, rare and atypical pathogens have been continuously detected, such as *Mycobacterium abscessus, Mycobacterium kansas*, etc. These pathogens may cause pneumonia, multiple-organ disorders and even acute respiratory distress syndrome (ARDS). Due to the limitations of current traditional pathogen detection methods in terms of sensitivity, detection speed and detection spectrum, rapid and accurate diagnosis of pneumonia pathogens are a big challenge (6, 7). Therefore, early and effective identification of the pathogens are essential for the precise treatment of pneumonia patients.

mNGS is the second-generation sequencing technology of metagenomics, which can identify bacteria, fungi, parasites, and viruses without much guidance from clinical experience. mNGS can identify the pathogens deeply and rapidly without culturing, and even have higher sensitivity than traditional cultivation methods (8). Another advantage of mNGS is the diversity of samples which could detect almost all pathogens in clinical samples (9) such as bronchoalveolar lavage fluid (BALF), tissue, sputum, pleural effusion, cerebrospinal fluid, pus, bone marrow, and nasal swabs (10–12), etc. Since the sensitivity and specificity of mNGS are less perturbative by the antibiotic treatment presently (13). mNGS may become a routine diagnostic test, partially replacing the traditional sputum culture method (14). However, the interpretation of the mNGS reports, especially the identification of pathogenic bacteria, colonizing bacteria, and the mixture of normal oral microbiota in respiratory tract samples in pneumonia patients need further study (15). The pathogenicity of microorganisms in different regions is generally different. There are few large-scale analysis research with respiratory samples studying the correlation between the detection efficiency of mNGS and the antibiotic therapy. Thus, our study aimed to explore the advantages of mNGS in the detection of pneumonia pathogens and its guiding significance for diagnosis and antibiotic treatment of CAP.

## Patients and Methods

### Patient Selection and Study Design

**Figure 1:**
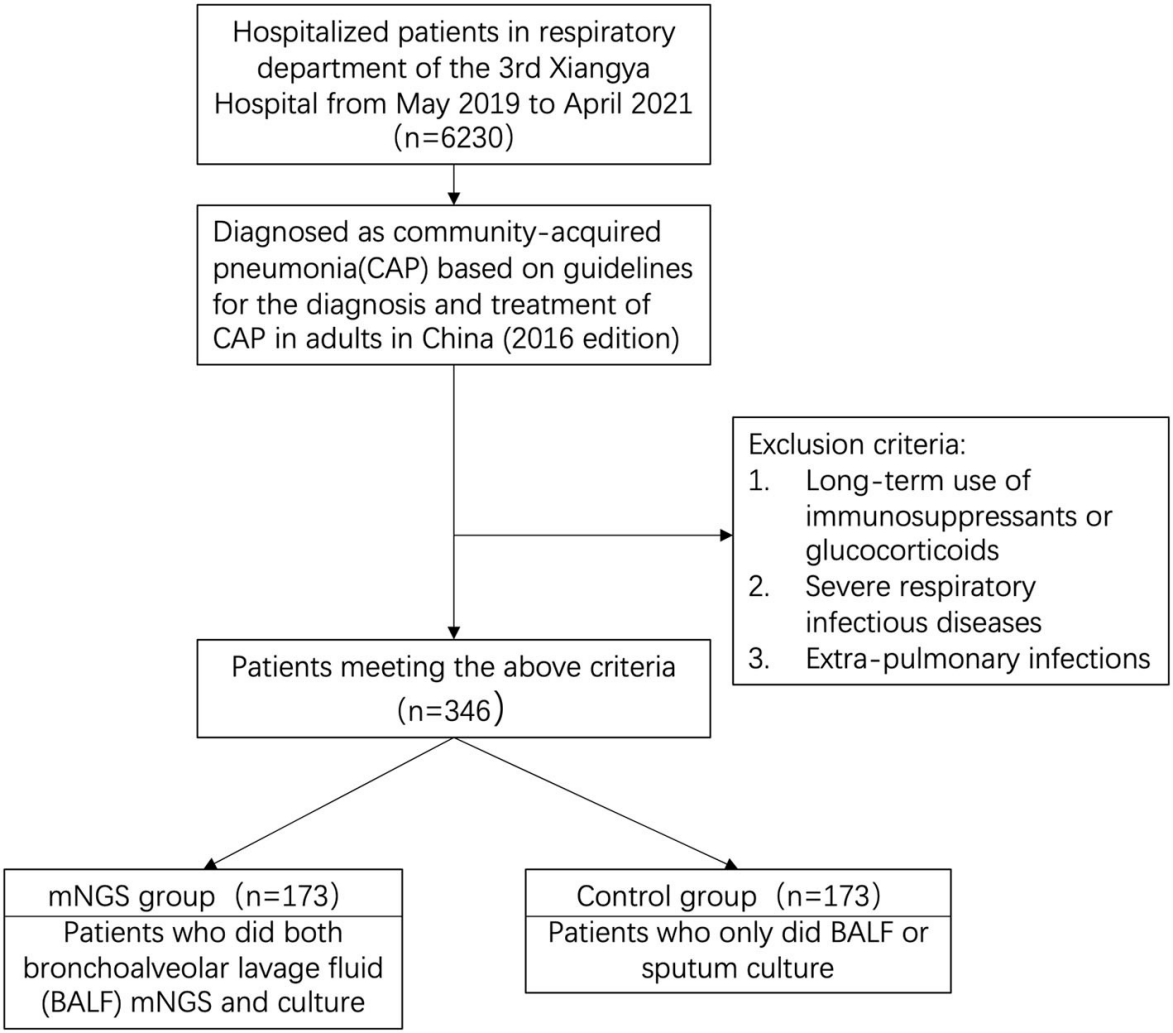
Flowchart of case selection. From 6230 cases, a total of 370 community-acquired pneumonia cases were selected for further analysis. Patients in mNGS group did the mNGS and BALF culture at the same time. Patients in control group only did the BALF or sputum culture. mNGS, metagenomic next-generation sequencing.

We retrospectively reviewed 346 cases diagnosed as CAP at the 3rd Xiangya Hospital of Central South University from May 2019 to April 2021. With our inclusion/exclusion criteria (Figure 1), 346 samples were included for analysis and categorized into two groups defined as mNGS group and control group. mNGS group was subjected to regular BALF culture as well as mNGS testing (ID: PRJNA756706, https://www.ncbi.nlm.nih.gov/sra/PRJNA756706) in a pairwise manner, and control group only did the BALF or sputum culture. This study was approved by Institutional Review Board of the 3rd Xiangya Hospital of Central South University (No. 21030).

### Sample Processing and Nucleic Acid Extraction

BALF were collected from patients according to standard procedures. DNA was extracted using a QIAamp^®^ UCP Pathogen DNA Kit (Qiagen) following the manufacturer's instructions and 600 μL of the processed specimens was mixed with glass beads of 0.1–0.2 mm diameter. A vortex mixer (Crystal, TX, United States) was used to disrupt the bacterial cell wall at 1,600 g for 10 min. The tubes were then heated at 99°C for 10 min before DNA extraction. Human DNA was removed using Benzonase (Qiagen) and Tween20 (Sigma) (16). The differential lysis method was used to remove host DNA. we first use physical hypotonic lysis and chemical lysis to break human cells, and then obtain microbial cells by enzymatic hydrolysis, followed by wall breaking and nucleic acid extraction. Total RNA was extracted with a QIAamp^®^ Viral RNA Kit (Qiagen) and ribosomal RNA was removed by a Ribo-Zero rRNA Removal Kit (Illumina). The concentration of extracted DNA/RNA was measured using a Qubit Fluorometer before library preparation. cDNA was generated using reverse transcriptase and dNTPs (Thermo Fisher).

### Library Preparation and Sequencing

Libraries were constructed for the DNA and cDNA samples using a Nextera XT DNA Library Prep Kit (Illumina, San Diego, CA) (17). The initial input of DNA is 5–100 ng. Firstly, DNA needs to be fragmented to obtain 150–250 bp inserts, followed by terminal repair and adapter connection, and finally, library amplification to construct a library that meets the requirements of sequencing. Library was quality assessed by Qubit dsDNA HS Assay kit followed by High Sensitivity DNA kit (Agilent) on an Agilent 2100 Bioanalyzer. Library pools were then loaded onto an Illumina Nextseq 550Dx sequencer for 75 cycles of single-end sequencing to generate ~20 million reads for each library. For negative controls, we also prepared PBMC samples with 10^5^ cells/mL from healthy donors in parallel with each batch, using the same protocol, and sterile deionized water was extracted alongside the specimens to serve as non-template controls (NTC).

### Bioinformatics Analyses

**Figure 2:**
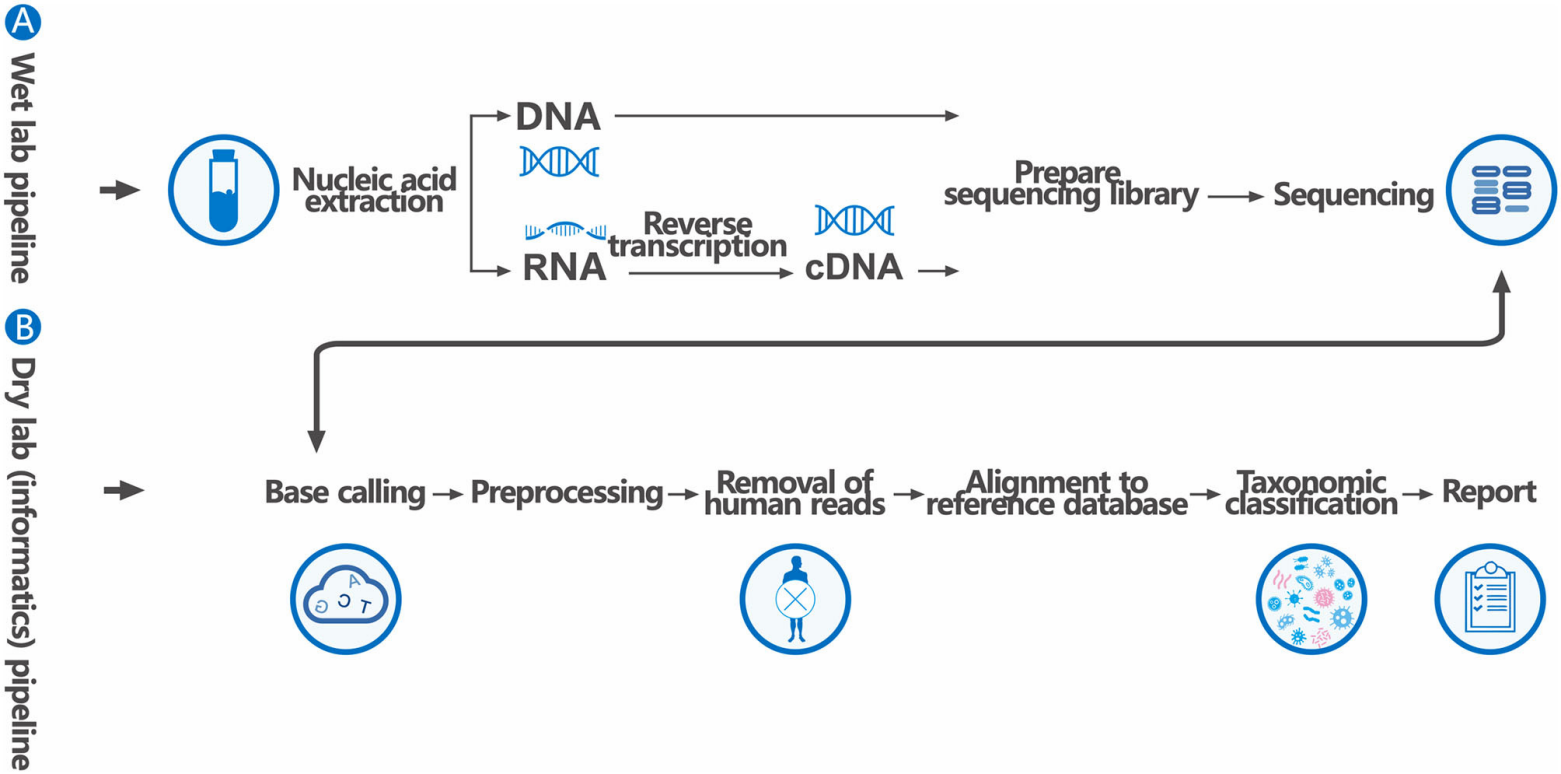
The generalized workflow of mNGS for clinical pathogen diagnosis. The workflow has two components: **(A)** a wet lab protocol in which samples are collected, processed, extracted for nucleic acids, prepared into a sequencing library, and sequenced, and **(B)** a dry lab computational pipeline that includes microbial identification, statistical analysis, and interpretation. The sequencing library may be targeted, undergo DNA amplification, or both.

Trimmomatic was used to remove low quality reads, adapter contamination, and duplicate reads, as well as those shorter than 50 bp (18). Low complexity reads were removed by Kcomplexity with default parameters (19). Human sequence data were identified and excluded by mapping to a human reference genome (hg38) using Burrows-Wheeler Aligner software. We designed a set of criteria similar to the National Center for Biotechnology Information (NCBI) criteria for selecting representative assembly for microorganisms (bacteria, viruses, fungi, protozoa, and other multicellular eukaryotic pathogens) from the NCBI Nucleotide and Genome databases. Pathogen lists was selected according to three references: (1) Johns Hopkins ABX Guide (https://www.hopkinsguides.com/hopkins/index/Johns_Hopkins_ABX_Guide/Pathogens), (2) Manual of Clinical Microbiology (https://www.clinmicronow.org/doi/book/10.1128/9781683670438.MCM), and (3) clinical case reports or research articles published in current peer-reviewed journals. The final database consisted of about 18,562 genomes. Microbial reads were aligned to database with SNAP v1.0beta.18. Virus-positive detection results (DNA or RNA viruses) were defined as the coverage of three or more non-overlapping regions on the genome. A positive detection was reported for a given species or genus if the reads per million (RPM) ratio, or RPM-r was ≥5, where the RPM-r was defined as the RPM_sample_ / RPM_NC_ (i.e., the RPM corresponding to a given species or genus in the clinical sample divided by the RPM in the NC/negative control). In addition, to minimize cross-species misalignments among closely related microorganisms, we penalized (reduced) the RPM of microorganisms sharing a genus or family designation, if the species or genus appeared in non-template controls. A penalty of 5% was used for species (12) (Figure 2).

### Statistical Analysis

Continuous variables were compared using the Mann–Whitney *U*-test; categorical variables were compared using the chi-square test. *P* < 0.05 was considered significant. Statistical analyses were performed using SPSS version 23.0 (SPSS, Inc., Chicago, IL, USA).

## Results

### Clinical Characteristics of Patients With CAP

Finally, a total of 346 patients with CAP was enrolled. One hundred and seventy-three patients were in the mNGS group, and the rest 173 patients were in the control group. The clinical characteristics of patients between these two groups were different as follows (Tables 1, 2). The rates of patients with comorbidities including hypertension, neoplastic, diabetic, renal, and cerebrovascular diseases were relatively lower in the mNGS group, but they did not reach statistical significance. However, the incidence of ARDS was significantly higher in the mNGS group (*P* = 0.021). Not unexpectedly, the average hospital stay was longer in the mNGS group (*P* = 0.001). But the empirical use of antibiotics in two groups were roughly the same. The acute physiology and chronic health evaluation (Apache) II score in two groups were not very different, either. The laboratory test results were 5–7 days after the mNGS and BALF or sputum culture did. Except for the platelet-to-lymphocyte (PTL) ratio and erythrocyte sedimentation rate (ESR), there were several significant differences between the two groups, indicating that the patients in mNGS group were relatively severer. Defined daily dose (DDD) represented the strength of antibiotic application and the antibiotics use density, which was higher in the mNGS group (*P* = 0.013) indicating the use of advanced antibiotics was more frequent.

**Table 1:** Demographic and baseline characteristics of patients.

**Characteristics**	**mNGS group (*n* = 173)**	**Control group (*n* = 173)**	***P***
Age, mean (range), years	60 (15–87)	64 (18–90)	0.045
Sex, female, *n* (%)	47 (27.2)	67 (38.7)	0.030
Hospital, mean (range), days	16 (1–66)	11 (0–70)	0.001
Comorbidity, *n* (%)	
Cardiovascular disease	34 (20.0)	35 (20.2)	1.000
Hypertension	43 (24.9)	51 (29.5)	0.398
Chronic obstructive pulmonary disease	19 (11.0)	17 (9.8)	0.860
Neoplastic disease	17 (9.8)	25 (14.5)	0.249
Diabetes	27 (15.6)	30 (17.3)	0.772
Kidney disease	8 (4.6)	12 (6.9)	0.490
Bronchiectasis	16 (9.2)	16 (9.2)	1.000
Cerebrovascular disease	22 (12.7)	26 (15.0)	0.641
ARDS, *n* (%)	49 (28.3)	30 (17.3)	0.021
On empiric antibiotics at time of sample collection, *n* (%)	173 (100)	171 (98.8)	0.499
Apache II score, mean ± standard deviation	14.0 ± 5.4	12.2 ± 6.4	0.051
30-day mortality, *n* (%)	14 (8.1)	12 (6.9)	0.839

*mNGS, metagenomic next generation sequencing; ARDS, acute respiratory distress syndrome*.

**Table 2:** Laboratory findings of patients.

	**mNGS group (*n* = 173)**	**Control group (*n* = 185)**	***P***
White blood cell count, x 10^9^/L	9.41 (0.56–57.27)	8.44 (1.68-29.47)	0.035
Percentage of neutrophils, %	77.9 (9.8–97)	72.5 (38.5–98.4)	0.001
Lymphocyte count, x 10^9^/L	1.07 (0.11–4.14)	1.28 (0.12–9.51)	0.005
NLR	11.18 (0.21–86.47)	9.05 (0.82–121.20)	0.001
PLR	282.82 (9.42–1925.00)	270.68 (6.00–1103.80)	0.803
Cre, umol/L	89 (30–855)	79 (26–527)	0.027
PCT, ng/mL	2.40 (0.01–75.57)	1.51 (0.01–70.62)	0.004
CRP, mg/L	82.65 (0.43–320.04)	50.14 (0.01–314.69)	0.001
ESR, mm/h	63.49 (2–120)	54.73 (2–120)	0.081
Ca^2+^, mmol/L	2.04 (0.99–2.49)	2.15 (1.15–3.34)	0.001
Albumin, g/L	30.0 (16.5–44.7)	32.5 (17.1–47.2)	0.001
DDD	116 (36–255)	109 (0–300)	0.013

*mNGS, metagenomic next generation sequencing; NLR, neutrophil-to-lymphocyte ratio; Cre, creatinine; PLR, platelet-to-lymphocyte ratio; DDD, defined daily dose; PCT, procalcitonin; CRP, C-reactive protein; ESR, erythrocyte sedimentation rate*.

*Data are presented as means (range)*.

### Comparison of Diagnostic Performance Between BALF mNGS and Culture

**Figure 3:**
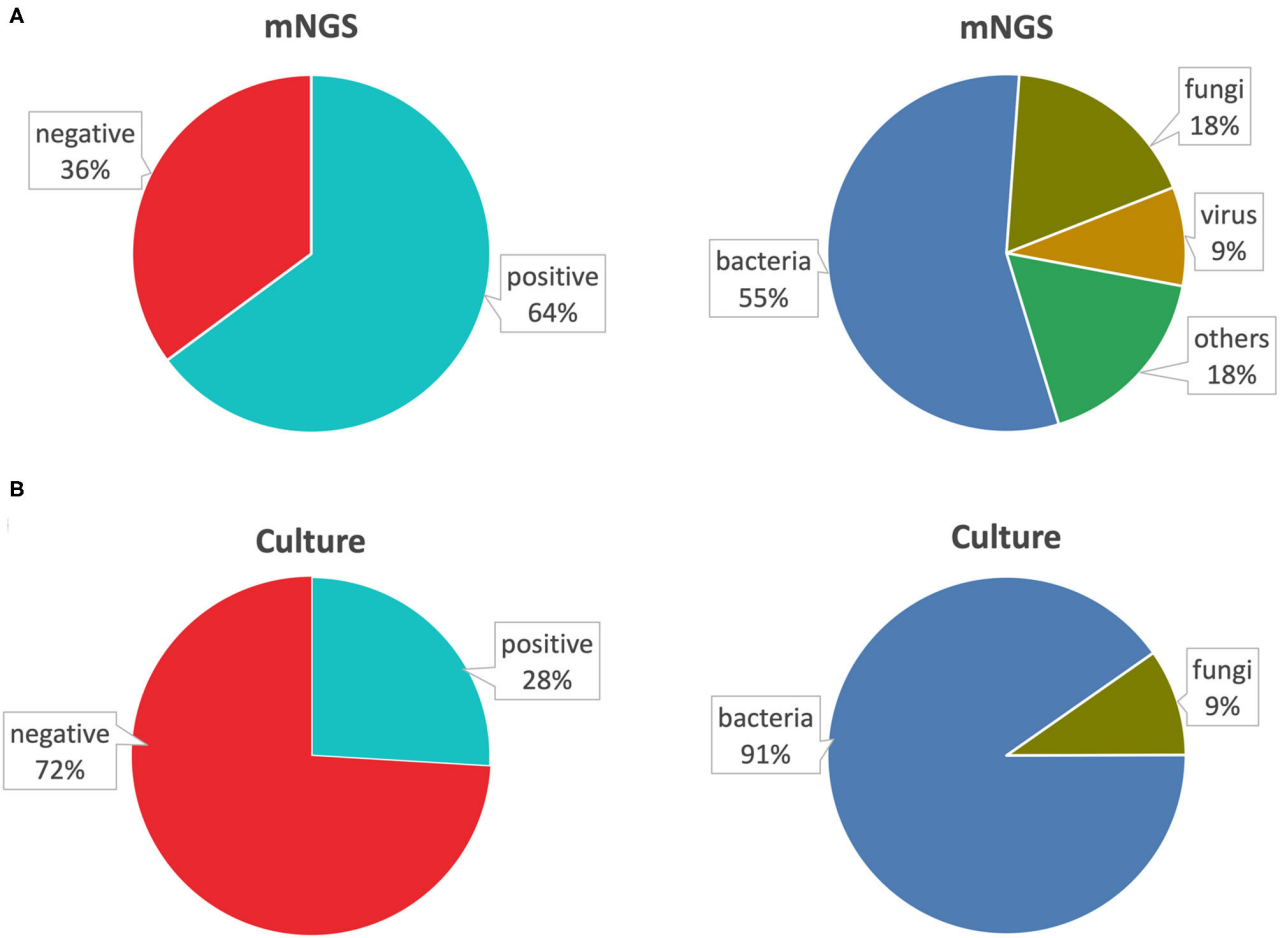
Comparison of positive rate of pathogens by BALF mNGS and culture. **(A)** The positive rate of mNGS and the types of pathogens detected. **(B)** The positive rate of BALF culture and the types of pathogens detected. BALF, bronchoalveolar lavage fluid; mNGS, metagenomic next-generation sequencing.

Within the mNGS group (both tested by mNGS and BALF culture), through BALF mNGS, 110 (64%) were detected positively with pathogens, and 20 (11%) were completely negative; 43 (25%) were detected with colonizing pathogens or contaminating pathogens, which we also considered as negative detection according to our experience. Furthermore, 92 (55%) were detected with bacteria, 30 (18%) with fungi, 15 (9%) with viruses, and 31 (18%) were detected with other special pathogens (Figure 3A). Conversely, BALF culture done at the same time with mNGS only showed 28% positive rate. Within this positive detection by BALF culture, only bacteria (91%) and fungi (9%) could be cultured, so the virus and other special pathogens could not be recognized by traditional cultures (Figure 3B). Thus, the sensitivity of mNGS was much higher than traditional microbiological culture. Within the mNGS group, we detected 24 cases of *Chlamydia psittaci* by mNGS, including eight female patients and 16 male patients, with the average age of 65 years old. In addition to *C. psittaci*, some of these patients were also infected with other pathogens, among which *Candida albicans* was the most (10/24). The reads of *C. psittaci* matched in these patients ranged from 16 to 270670. The relative abundance refers to the proportion of pathogen in the same type of microorganism, and the relative abundance of *Chlamydia* was: 0.1–97.9%. A higher relative abundance indicated a higher proportion of the species in the sample. Relative abundance was only a parameter that indicates the amount of pathogens, and it could not be directly judged whether it was pathogenic or not based on the value of relative abundance. Therefore, we did not compare the relative abundance of detected pathogens. In the process of clinical diagnosis, the value of relative abundance was only for reference. Sixteen out of 24 patients adjusted the use of antibiotics based on the reports of mNGS. The white blood cells and neutrophils of most patients did not increase significantly, but the increase of PCT and D-dimer and the decrease of blood calcium were related to the severity of the condition (Supplementary Table 1).

### Comparison of Pathogens Detected by BALF mNGS and Culture

**Figure 4:**
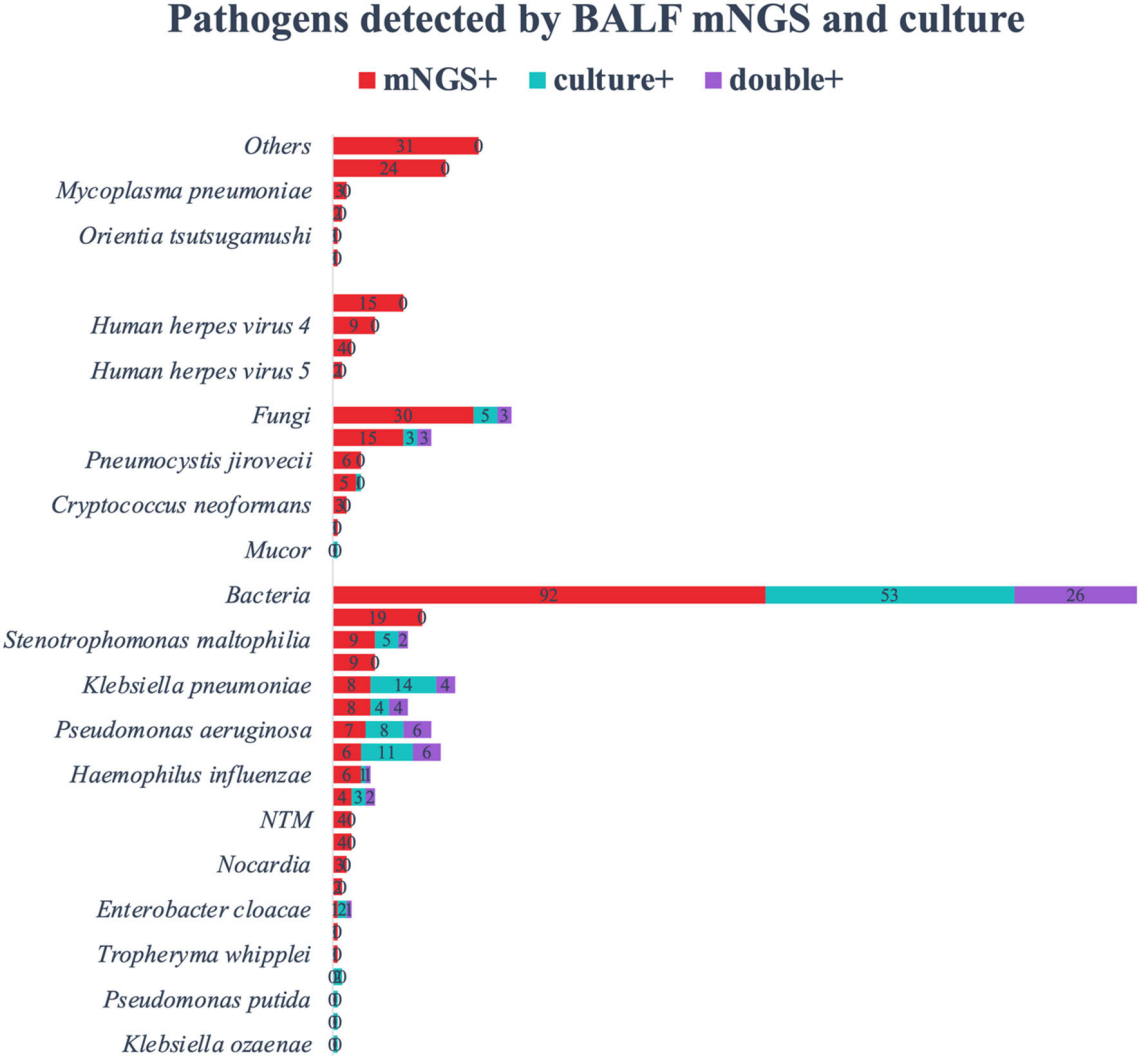
The overlap of positivity between BALF mNGS and BALF culture. Bacteria were the most detected, followed by fungi, special pathogens, and viruses. The positive rate of BALF culture in detecting *Klebsiella pneumoniae* and *Acinetobacter baumannii* was higher than that of mNGS. *Chlamydia psittaci* was the most detected by mNGS, followed by *Haemophilus parainfluenzae* and *Candida albicans*. All viruses, special pathogens, MTB and NTM were only detected by mNGS, and the BALF culture of these pathogens were negative. BALF, bronchoalveolar lavage fluid; mNGS, metagenomic next generation sequencing; MTB, *Mycobacterium tuberculosis*; NTM, *nontuberculous mycobacteria*.

**Figure 5:**
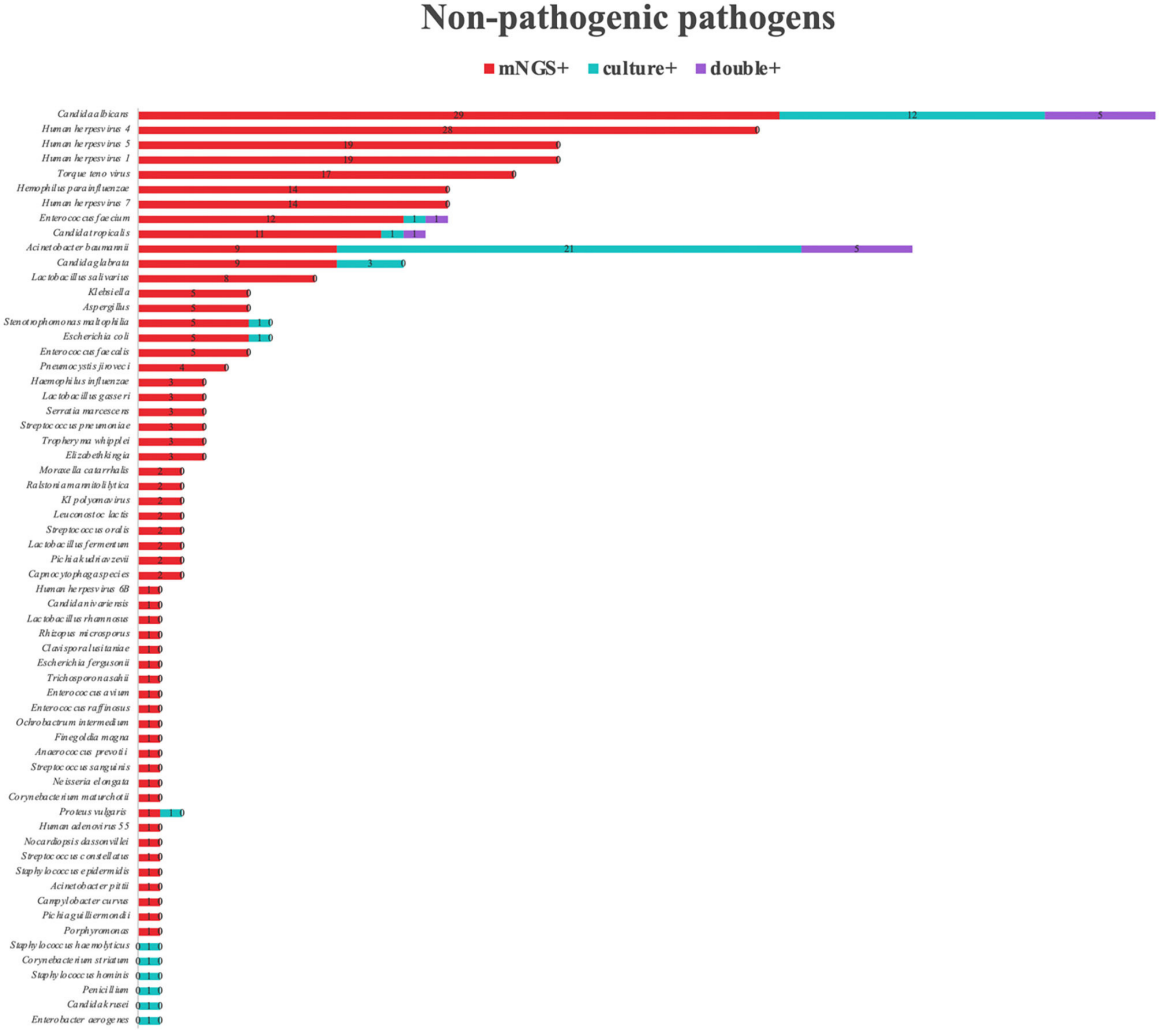
Non-pathogenic pathogens detected by mNGS. A total of 64 different non-pathogenic pathogens were detected in the mNGS group. *Human herpesvirus 5, Candida albicans, Human herpesvirus 4* are the most common non-pathogenic pathogens detected by mNGS. *Acinetobacter baumannii, Candida albicans, Candida glabrata* are the most common non-pathogenic pathogens detected by sputum culture. mNGS, metagenomic next-generation sequencing.

Among the 168 kinds of morbigenous microorganism, *C. psittaci* (24/168) was the most detected pathogen by mNGS, followed by *Hemophilus parainfluenzae* (19/168). We also detected virus (15/168), and other special pathogens (31/168). The positive rate of mNGS for pathogenic bacteria was almost twice than that of BALF culture (Figure 4). Except for *K. pneumoniae* and *Acinetobacter baumannii*, the positive rate for mNGS of other pathogens were relatively higher. Not only that, *Haemophilus parainfluenzae, Mycobacterium tuberculosis, Nocardia, Legionella, Streptococcus parasanguis*, and *Tropheryma whipplei* were only detected by mNGS, and the BALF cultures of these microorganism were all negative. The positive rate of mNGS for fungi was seven times than that of BALF culture, and *C. albicans* was one of the most detected fungi in BALF culture. For *Pneumocystis jirovecii, Aspergillus*, and *Cryptococcus*, the positive rate of mNGS was much higher than that of BALF culture. Viruses and some special pathogens can only be detected by mNGS. *Human herpesvirus 1, 4*, and *5* were only considered pathogenic when the reads and abundance was relatively high. mNGS can detect rare pathogens such as *Orientia tsutsugamushi* and *Leptospira interrogans*, which was of great guiding significance for antibiotic adjustment. In addition, we also counted other pathogens in the mNGS report of 173 patients that we thought were not pathogenic (Figure 5). Among the non-pathogenic pathogens, *C. albicans* was the most common. But the *A. baumannii, K. pneumoniae*, etc. were also included which were “usually” considered to cause pneumonia. As there was no detailed uniform criteria or authoritative guide for the interpretation of mNGS reports, we always determined whether the pathogens were pathogenic, colonized, or contaminated based on clinical experience, patient's imaging findings and inflammation indicators.

### The Influence of mNGS on Treatment and Prognosis

**Figure 6:**
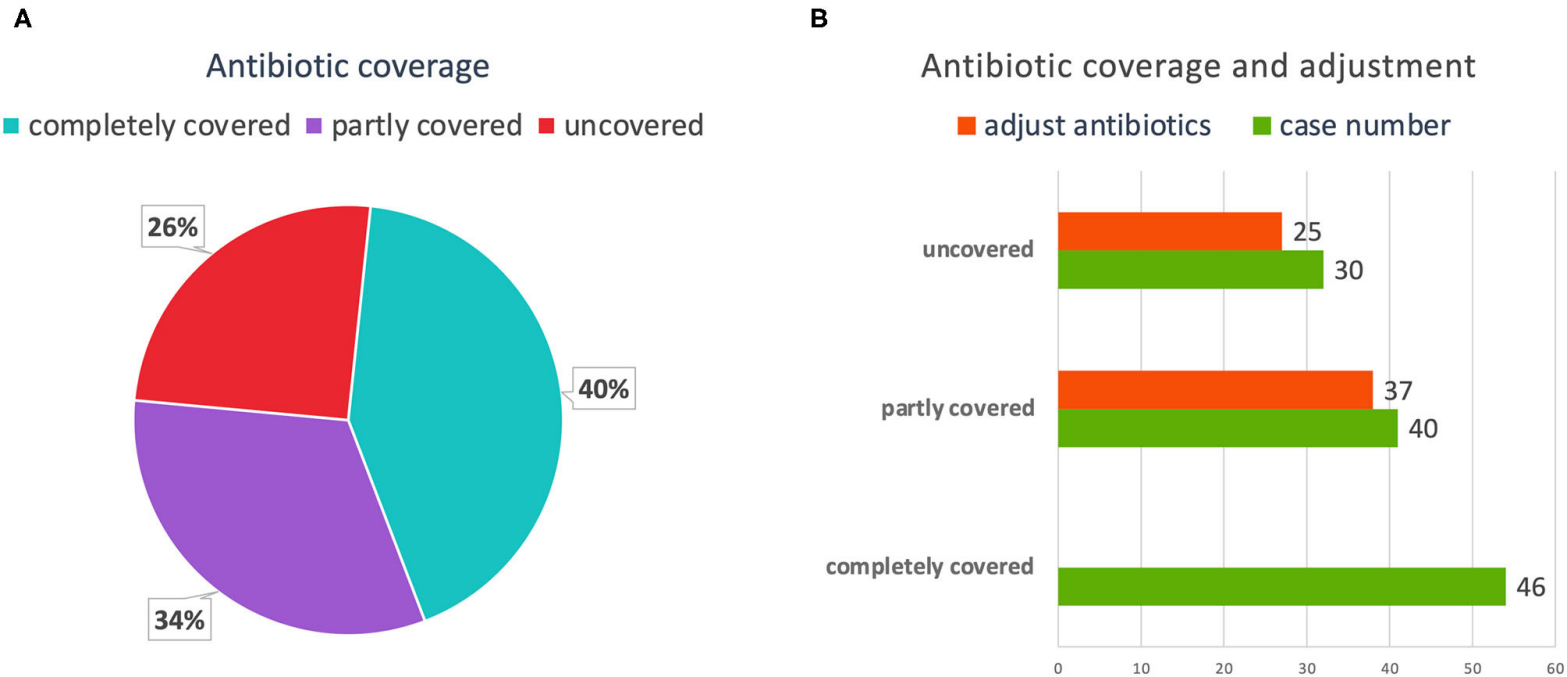
The coverage and adjustment of antibiotics in the mNGS group. **(A)** Among patients in the mNGS group, complete antibiotic coverage was 54 (43%); partial coverage was 41 (32%); and no coverage was 32 (25%). **(B)** Antibiotics were adjusted for 38 partially covered patients, and for 27 uncovered patients. mNGS, metagenomic next-generation sequencing.

The correct use of antibiotics was extremely important for the treatment of CAP. Among the 116 patients with pathogen-positive pneumonia, 46 (40%) cases were completely covered by antibiotics before the pathogens were detected. These 46 cases were not adjusted for antibiotics after the pathogens were detected. Forty (34%) cases were partially covered by antibiotics before the pathogens were detected. After the pathogens were detected, 37 cases adjusted their antibiotics, and three cases did not adjust, of which one case were transferred to specialist hospitals for further treatment, and two cases died. Thirty (26%) cases were not covered by antibiotics at all, of which 25 cases were adjusted after the pathogens were detected, and the remaining five cases were transferred to other hospitals or death (Figure 6). All the pathogens detected by mNGS and culture, and the details of antibiotics therapy had been listed in Supplementary Table 2. As for pathogens were not detected or pathogens that were colonized and contaminated, we could only rely on laboratory test results, imaging findings, and empirical treatment to adjust antibiotics.

## Discussion

We retrospectively reviewed 346 CAP cases. Based on the patients' clinical characteristics and inflammatory indices, the patients in the mNGS group were relatively severer in our study, including a higher incidence of ARDS, WBC, PCT, neutrophils, ESR elevation, and peripheral blood calcium concentration decrease. We suggested that mNGS would be recommended to patients with more complex and severer conditions to accurately identify the pathogens and timely adjust antibiotics. The DDD reflects the density of antibiotic application. The patients in the mNGS group treated more antibiotics because their conditions were more serious and complicated. However, we have also controlled it at a relatively low level which was benefited from the timely detection of pathogens by mNGS. The patients received timely targeted anti-infection treatment, and most of them had better prognosis. In addition, although patients in the mNGS group were with severer conditions, the 30-day mortality rate was basically the same as that in the control group, which indicated that the early diagnosis and promptly treatment of patients through mNGS could reduce the mortality. Moreover, mNGS can detect many pathogens that cannot be detected by traditional microbiological culture. During the incipient stage of the COVID-19 pandemic, mNGS supplied a quick and accurate identification for pathogenic virus (20).

In the past, we thought that *C. psittaci* was relatively rare. However, we detected 24 patients infected with *C. psittaci* through mNGS. Most of the cases were also accompanied by the infection of other pathogens (*C. albicans* was the top one), but many of the pathogens were the microbiota in oropharynx and colonized bacteria in respiratory tracts. Meanwhile, we found that after mNGS had been fully applied in our clinical practice from the year 2018, the incidence of *C. psittaci* pneumonia had been greatly increased, which meant that *C. psittaci* was widespread in the past, but it was difficult to be detected. Although miost patients infected with *C. psittaci* have severe pneumonia, clinicians can adjust the dosage and classes of antibiotics in time according to mNGS reports, and the overall outcome of the patients is better. Previous studies also confirmed that mNGS could improve the outcome of patients with severe pneumonia of *C. psittaci* and played a positive role in diagnosis and treatment of CAP, as well as adjustment of antibiotics (21).

Traditionally, the detection of tuberculosis could only be based on methods such as acid-fast staining of sputum smears, culture of *M. tuberculosis*, and interferon gamma release assay (IGRA), etc. These methods have disadvantages such as low sensitivity, low positive rate, time-consuming, and not direct enough. However, mNGS can detect as low as 1–2 reads of *M. tuberculosis* which provides an opportunity for patients to be transferred to specialist hospitals for tuberculosis therapy in time. In the past few decades, the detection of *O. tsutsugamushi* and *L. interrogans* could only rely on microscopic examination, but mNGS can currently detect these special pathogens sensitively, which provides a good guidance for clinicians to adjust treatment protocols. Among the 116 pathogen-positive cases, the positive rate of mNGS for bacteria and fungi were significantly higher than that of BALF culture, additionally mNGS can detect viruses and special pathogens sensitively.

Due to regional differences, the pathogens of our CAP cases were quite different from those in northern China. Chen et al. (22) reported that the bacteria such *as Citrobacter freundii, Salmonella enterica*, and *Aeromonas hydrophila* detected by mNGS are common pathogens in CAP. But those bacteria were not detected in any of our cases, which was possibly because the patients in Peking University People's Hospital was more complicated since the difficult and complicated patients around the country would like to go there for further treatment, so the pathogenic pathogens could have a multiple source.

Among the non-pathogenic pathogens detected by mNGS, *Human herpesvirus 4, 1*, and *5* were the most common viruses. These viruses can be latent in the host when the patient is immunocompetent (23). Most studies believed that they had no pathogenic significance when detected in BALF. But when the patient's immune function was low or suppressed, these viruses will be pathogenic (24). *C. albicans* was also detected relatively frequently, but pneumonia with *C. albicans* was relatively rare (25), and most of it were hematogenous dissemination. Additionally, *Enterococcus faecium* and *Enterococcus faecalis* pneumonia were also very rare (26). When interpreting the mNGS reports, some clinicians thought that *C. albicans* was pathogenic. We believed that anti-*C. albicans* treatment must be determined based on the patient's imaging findings and inflammatory indicators. *Torque teno virus* is widely present in the human body, animals, air, and solid surfaces (27), which caused pneumonia only when patients were under immunosuppression condition (28). Therefore, the positive report of *Torque teno virus* may be sample contamination or in immunosuppression condition. When the reads of *H. parainfluenzae* are not high, it is necessary to comprehensively determine whether it is pathogenic based on clinical features, infection sites, inflammation indicators, and lung imaging findings. Some cases of *A. baumannii* and *K. pneumoniae* are in-hospital infection (29, 30). Patients with long-term use of antibiotics and longer hospital stays are prone to in-hospital infection. The impaired intestinal barrier, long-term use of acid inhibitors, long-term bed rest, and nasogastric reflux will cause bacterial translocation (31). The primary lesions of these patients are not in the lungs. Therefore, *A. baumannii* and *K. pneumoniae* detected in the BALF of these patients cannot be regarded as the pathogenic bacteria for pneumonia. The use of mNGS in patients with immunosuppression not only identifies pathogens, but also reflects the patient's immune status and microbiota distribution. A small number of cases in which the detection was negative with mNGS, but the BALF culture was positive, which was possibly because: (1) the specimen may have been contaminated. For example, the positive *Aspergillus* BALF culture may be caused by contamination. (2) The low pathogens load in the specimen. Under suitable culture conditions, active micro bacteria or fungi could also be cultured, but micro pathogens could not reach the minimum threshold of mNGS detection. (3) Detection of some fungi requires breaking the cell wall to obtain DNA, but conventional sputum culture does not. Therefore, if this process was badly handled, negative results of mNGS may occur. Nevertheless, mNGS showed significant advantages for detecting fastidious bacteria.

Normal sputum culture takes 5–7 days. *M. tuberculosis* culture even takes about 6 weeks. But mNGS can detect pathogens within 48 h and even shorter, which greatly improves the timeliness of treatment. In the use of antibiotics, mNGS can be used to determine whether the current antibiotic therapy covered the pathogens and the reads of pathogens detected by mNGS will guide clinicians to adjust the dosage of antibiotics. Moxifloxacin was the common choice for the severe CAP regularly. However, after the *C. psittaci* was detected by mNGS, we adjusted the moxifloxacin to the doxycycline. Additionally, after the rare pathogens such as *L. interrogans* detected, adjusted to penicillin G was essential for the initial treatment stage. After the fungi detected by mNGS, we should take a comprehensive consideration between the specific reads and abundance of the pathogens to determine whether use the antifungal agents such as fluconazole or voriconazole.

However, there are still some disadvantages of mNGS. Due to the high sensitivity of mNGS, some colonized and contaminated pathogens will also be detected. Because of the differences in the ability of clinicians to interpret the mNGS reports, it may lead to the abuse of antibiotics. The mNGS performed during the early stage of hospitalization could clarify what the main pathogen is. After a period of hospitalization, patients with underlying diseases or immunocompromised patients may have in-hospital infections or colonization of some special bacteria, which will affect the interpretation of mNGS results. Besides, some clinicians are not strict enough on the indications for mNGS, which leads to a waste of medical resources. Otherwise, some laboratories will delete background bacteria, but occasionally the main pathogenic bacteria may be deleted. mNGS has some shortcomings, and it is not a routine pathogen detection method in the guideline. However, due to the large population and a vast extent of land, medical resources are not evenly distributed to a certain extent. The technology of pathogen detection in some remote areas is poor, and even the PCR technology for single pathogens is lacking. Therefore, it is necessary to use mNGS to detect pathogens in an appropriate and timely manner.

Therefore, we suggest that mNGS would be mainly used: (1) the infection is complicated and severe; (2) pathogens which are hard to detected by traditional microorganism test or culture, such as *M. tuberculosis* and *Cryptococcus*. We need to improve clinicians' ability of interpretation of mNGS reports to prevent the abuse of antibiotics caused by mNGS.

The limitation in our study is that this is a single-center retrospective study with a relatively small sample size, thus there was selection bias. Since we tended to compare the positive rate of mNGS and BALF culture, as well as the characteristics of the detected pathogens, the 173 control patients who only underwent BALF or sputum culture were not used for subsequent analysis of pathogen characteristics. As the antibiotic coverage of pneumonia patients was close to 100%, the positive rate of BALF or sputum culture would be affected by antibiotics, possibly resulting in a relative higher positive rate of mNGS. All mNGS reports were interpreted by a senior clinician, so the consistency was ensured.

## Conclusion

The positive rate of mNGS for pathogens in patients with CAP was higher than that of traditional BALF culture. mNGS can detect pathogens in a more timely and accurate manner and assist clinicians to adjust antibiotics in time. With available indications, we recommend mNGS for the complementary diagnosis of severe or complicated pneumonia.

## Data Availability Statement

The data presented in the study are deposited in the SRA repository, accession number PRJNA756706.

## Ethics Statement

The studies involving human participants were reviewed and approved by Institutional Review Board of the 3rd Xiangya Hospital of Central South University (No. 21030). The patients/participants provided their written informed consent to participate in this study. Written informed consent was obtained from the individual(s) for the publication of any potentially identifiable images or data included in this article.

## Author Contributions

The study was conceived, designed, and supervised by CL and QZ. Statistical analyses were performed by YZ. Clinical data collection was done by HL. Sample collection was done by SS. Manuscript was written by HL and YZ. Figures and tables were drawn by GC. mNGS testing data was uploaded by FC. Manuscript editing was done by JW, HL, and YZ contributed equally to this study. All authors contributed to the article and approved the submitted version.

## Funding

This study was supported by grants from the National Natural Science Foundation of China (81700658), the Hunan Provincial Natural Science Foundation-Outstanding Youth Foundation (2020JJ3058), the Key Research and Development Program of Hunan Province (2020DK2001), and the Science and Technology Innovation Project of Hunan Province (2020SK53608).

## Conflict of Interest

FC was employed by Vision Medicals Co. Ltd (Guangzhou, China). The remaining authors declare that the research was conducted in the absence of any commercial or financial relationships that could be construed as a potential conflict of interest.

## Publisher's Note

All claims expressed in this article are solely those of the authors and do not necessarily represent those of their affiliated organizations, or those of the publisher, the editors and the reviewers. Any product that may be evaluated in this article, or claim that may be made by its manufacturer, is not guaranteed or endorsed by the publisher.
