# Supplementary material for: Diagnostic Significance of Metagenomic Next-Generation Sequencing for Community-Acquired Pneumonia in Southern China
Source: Front Med (Lausanne). 2022 Feb 15;9:807174. doi: 10.3389/fmed.2022.807174 (PMC8885724; doi:10.3389/fmed.2022.807174)
Supplement: Supplementary file 1 [file Table_1.DOCX]

| Supplementary table 1. CAP patients infected with Chlamydia psittaci for mNGS and conventional laboratory-based tests | | | | | | | | | | | | | |
| --- | --- | --- | --- | --- | --- | --- | --- | --- | --- | --- | --- | --- | --- |
|  | | | | | | | | | | | | | |
| Patient ID | Sex | Age | mNGS results | | | Severe pneumonia | Antibiotic coverage | WBC（10^9^/l） | NGP（%） | Ca^2+^（mmol/l） | D-dimer（mg/l） | PCT（ng/ml） | Outcome |
|  |  |  | **Pathogen** | **The relative abundance of *Chlamydia*（%）** | **Reads of *Chlamydia psittaci*** |  |  |  |  |  |  |  |  |
| 01 | Female | 54 | *Chlamydia psittaci; Lactobacillus salivarius; Candida albicans; Candida glabrata* | 0.1 | 56 | Yes | Yes | 5.18 | 85.5 | 1.92 | 1.56 | 2.43 | Cure |
| 02 | Female | 56 | *Chlamydia psittaci; Candida albicans; Human gammaherpesvirus 4* | 41.8 | 5235 | Yes | No | 5.63 | 95.1 | 1.82 | 2.44 | 4.1 | Cure |
| 03 | Felmale | 70 | *Chlamydia psittaci* | 29.1 | 113 | Yes | No | 17.62 | 94.8 | 1.79 | 4.74 | 10.29 | Cure |
| 04 | Male | 76 | *Chlamydia psittaci; Staphylococcus aureus; Acinetobacter baumannii; Candida albicans* | 12.9 | 369 | Yes | No | 7.02 | 87 | 1.98 | 3.21 | 0.8 | Death |
| 05 | Male | 51 | *Chlamydia psittaci; Human gammaherpesvirus 4* | 47.4 | 277 | Yes | Yes | 18.96 | 93.7 | 2.32 | 1.78 | 4.72 | Cure |
| 06 | Male | 78 | *Chlamydia psittaci; Enterococcus faecium; Candida albicans; Candida tropicalis* | 92.61 | 440 | Yes | No | 15.19 | 95.7 | 1.96 | 23.62 | 18.04 | Cure |
| 07 | Male | 78 | *Chlamydia psittaci; Candida albicans* | 2.0 | 180 | Yes | No | 7.2 | 90.5 | 2.16 | 2.385 | 0.4 | Cure |
| 08 | Male | 76 | *Chlamydia psittaci; Enterococcus faecium; Haemophilus influenzae; Human gammaherpesvirus 4; Human betaherpesvirus 7* | 1.6 | 3161 | Yes | No | 3.83 | 89.9 | 1.70 | 1.5 | 9.75 | Cure |
| 09 | Male | 70 | *Chlamydia psittaci; Tropheryma whipplei; Human gammaherpesvirus 4* | 0.1 | 47 | Yes | No | 5.18 | 76.3 | 1.95 | 1.78 | 0.51 | Cure |
| 10 | Male | 75 | *Chlamydia psittaci* | 42.8 | 50 | No | Yes | 7.82 | 85.8 | 2.03 | 1.23 | 0.85 | Cure |
| 11 | Male | 52 | *Chlamydia psittaci* | 97.91 | 3240 | Yes | No | 8.27 | 86.5 | 2.06 | 7.42 | 43.73 | Cure |
| 12 | Famale | 69 | *Chlamydia psittaci; Enterococcus faecalis; Nocardia asiatica; Haemophilus parainfluenzae; Candida albicans; Human alphaherpesvirus 1* | 84.3 | 208359 | Yes | No | 12.22 | 94.6 | 2.06 | 17.24 | 5.19 | Cure |
| 13 | Female | 61 | *Chlamydia psittaci; Candida albicans; Human gammaherpesvirus 4* | 0.1 | 79 | No | Yes | 4.38 | 80.5 | 1.93 | 1.26 | 0.24 | Cure |
| 14 | Male | 52 | *Chlamydia psittaci Human gammaherpesvirus 4* | 41.8 | 102 | Yes | No | 10.8 | 92.8 | 1.8 | 2.1 | 3.27 | Cure |
| 15 | Male | 59 | *Chlamydia psittaci；* | 72.4 | 274 | No | No | 5.6 | 89.4 | 2.00 | 1.65 | 0.72 | Cure |
| 16 | Female | 72 | *Chlamydia psittaci; Haemophilus parainfluenzae; Tropheryma whipplei; Torque teno virus* | 96.6 | 270670 | No | Yes | 24.91 | 94.7 | 2.1 | 4.05 | 0.8 | Cure |
| 17 | Male | 57 | *Chlamydia psittaci; Human gammaherpesvirus 4* | 96.5 | 5805 | Yes | No | 17.36 | 95.3 | 1.06 | 1.78 | 18.11 | Death |
| 18 | Male | 61 | *Chlamydia psittaci；* | 5.45 | 16 | Yes | No | 3.3 | 66 | 1.74 | 0.45 | 0.61 | Cure |
| 19 | Female | 69 | *Chlamydia psittaci；* | 80.6 | 158 | Yes | Yes | 12.86 | 87.8 | 2.04 | 4.15 | 35.29 | Cure |
| 20 | Male | 80 | *Chlamydia psittaci; Candida albicans; Human alphaherpesvirus 1* | 39.5 | 23889 | Yes | No | 9.11 | 93.9 | 1.81 | 4.32 | 1.56 | Cure |
| 21 | Male | 43 | *Chlamydia psittaci; Candida albicans; Human alphaherpesvirus 1* | 2.2 | 559 | Yes | No | 3.29 | 79.3 | 1.95 | 2.5 | 0.46 | Cure |
| 22 | Female | 64 | *Chlamydia psittaci；* | 72.9 | 827 | Yes | Yes | 5.43 | 75.1 | 1.79 | 2.37 | 0.26 | Cure |
| 23 | Male | 70 | *Chlamydia psittaci；* | 83.0 | 20847 | Yes | Yes | 9.51 | 96.5 | 2.18 | 11.73 | 18.44 | Cure |
| 24 | Male | 67 | *Chlamydia psittaci; Candida albicans* | 71.4 | 13923 | Yes | No | 4.99 | 88.6 | 1.83 | 3.95 | 1.29 | Cure |

Abbreviations: mNGS, metagenomic next-generation sequencing; NGP, neutrophilic granulocyte percentage; WBC, white blood cells; PCT, procalcitonin.
